# High performance of point-of-care rapid tests for advanced HIV disease diagnosis by lay providers in Malawi: Results from a prospective diagnostic accuracy study supporting decentralized advanced HIV disease screening

**DOI:** 10.1371/journal.pone.0340955

**Published:** 2026-06-18

**Authors:** Agness Thawani, Ethel Rambiki, Jacqueline Huwa, Layout Gabriel, Aubrey Kudzala, Christine Kiruthu-Kamamia, Pachawo Bisani, Shameem Buleya, Nathan P. Ford, Cheryl C. Johnson, Ajay Rangaraj, Robert Luo, Celine Lastrucci, Busisiwe Msimanga, Rose Nyirenda, Bilaal Wilson Matola, Andreas Jahn, Claudia Wallrauch, Rachael Burke, Tom Heller

**Affiliations:** 1 Lighthouse Trust, Lilongwe, Malawi; 2 International Training and Education Center for Health, University of Washington, Seattle, Washington, United States of America; 3 Global HIV, Hepatitis and STI Programmes, World Health Organization, Geneva, Switzerland; 4 Department of HIV and AIDS, Ministry of Health, Malawi; 5 LMU University Hospital Munich, Division of Infectious Diseases and Tropical Medicine, Munich, Germany; 6 London School of Hygiene & Tropical Medicine, United Kingdom of Great Britain and Northern Ireland; Gulu University, UGANDA

## Abstract

CD4 testing is essential for identifying people with advanced HIV(AHD) disease and enabling delivery of the recommended diagnostic package including serum cryptococcal antigen (CrAG)and urine lipoarabinomannan(LAM) testing and prophylaxis. Suboptimal access to CD4 testing remains a major barrier to the scale-up of advanced HIV disease services, and there is limited evidence to inform strategies for improving access within constrained health systems. Although task-sharing for advanced HIV disease is being promoted, evidence on the performance of these diagnostics when delivered by lay providers in routine program settings remains limited. Lay providers (HIV diagnostic assistants) might be able to perform CD4 testing, serum cryptococcal antigen (CrAg) and urine lipoarabinomannan (LAM) tests using lateral flow assays (LFAs). We conducted a prospective diagnostic accuracy study comparing VISITECT® CD4 lateral flow assay results performed by HDAs and laboratory technicians, using paired quantitative PIMA CD4 results performed by nurses as the reference standard. We also compared serum cryptococcal antigen and urine lipoarabinomannan test results performed by HDAs and nurses. Implementation costs were estimated to assess the potential efficiency of task-sharing. We recruited 308 participants, the median CD4 was 248 cells/mm^3^; and 115 participants (37.3%) had values below 200 cells/mm^3^. Sensitivity and specificity for determining CD4 below 200 cells/mm^3^ using the VISITECT® CD4 LFA operated by HDAs were 94.8% (95% CI: 89.1–97.6%) and 92.2% (95% CI: 87.6–95.2%), respectively. HDAs achieved higher sensitivity and specificity than laboratory technicians. Subsequent serum-CrAg and urine-LAM test performed by HDAs and nurses showed an agreement of 98.1% (κ = 0.74) and 98.1% (k = 0.85), respectively. Incremental cost per CD4 test was US$8.69 when performed using the PIMA® CD4 quantitative device by a nurse and US$5.24 when performed using the VISITECT® CD4 semi-quantitative lateral flow assay by HDAs (2024 US dollars). Trained lay providers can accurately perform CD4, urine TB-LAM, and serum cryptococcal antigen testing. Our findings support task-sharing for decentralized advanced HIV disease testing; CD4 lateral flow assay testing is particularly suitable for peripheral health facilities that rely on lay providers and lack reliable electricity and laboratory infrastructure. This approach could play a critical role in expanding access to advanced HIV disease services.

## Introduction

Despite the remarkable progress with antiretroviral therapy (ART) rollout in sub-Saharan Africa, approximately one quarter to one third of people living with HIV (PLHIV) presenting to care continue to have advanced HIV disease (AHD) [1–5]. In 2017, the World Health Organization (WHO) recommended a package of care for AHD, which included screening, treatment or prophylaxis for major opportunistic infections, rapid initiation of ART, and intensified adherence support for all patients presenting with AHD [6]. AHD remains a leading cause of hospital admissions among PLHIV globally [7]. Enhanced infection prophylaxis has been shown to reduce mortality in severely immunosuppressed adults and older children initiating ART [8]. Most reporting countries have fully (75%) or partially (17%) adopted this recommended AHD package of care [9].

According to WHO and Malawi national guidelines, screening for advanced HIV disease using CD4 testing is prioritized for people living with HIV who are newly initiating ART, re-initiating ART after treatment interruption, have suspected or confirmed treatment failure (including high viral load), or who are hospitalized, seriously ill, or clinically unstable. CD4 testing plays a key role in the identification of AHD due to the large proportion of patients with advanced immune suppression who present with few or no clinical symptoms. People with AHD are at high risk of rapid disease progression and development of opportunistic infections [6,7]. A CD4 cell count of ≤200 cells/mm3 should trigger testing for disseminated TB using urinary lipoarabinomannan antigen (urine-LAM) and cryptococcal antigen in serum (serum-CrAg) to screen for Cryptococcal meningitis (CM) using lateral flow assays (LFAs) [6].

Despite policy recommendations, there is suboptimal uptake of AHD services primarily due to limited access to CD4 testing at, or near, the point-of-care (POC). Access to CD4 testing represents the main bottleneck in the AHD cascade, hindering early identification, further investigation, and treatment.

We aimed to evaluate the performance of the VISITECT® CD4 semi-quantitative lateral flow assay (LFA) test conducted by HIV diagnostic Assistants (HDAs). The VISITECT® CD4 LFA, manufactured by Omega Diagnostics, is a semi-quantitative, point-of-care assay designed to identify individuals with CD4 counts below 200 cells/µL, enabling rapid detection of advanced HIV disease without the need for laboratory-based platforms. The test involves multiple timed steps and visual interpretation of bands, which may introduce user-dependent variability. This diagnostic rapid test has been assessed for diagnostic accuracy in a multi country study that included Malawi, Zimbabwe, and the DRC and showed good performance with high sensitivity ≥95% on both finger prick and venous blood and specificity of 77.2% and 81.9% respectively. High usability of the VISITECT® CD4 was also observed in this multi-country evaluation [10].

In this evaluation, the primary objective was to assess the diagnostic accuracy and feasibility of VISITECT® CD4 LFA testing when performed and interpreted by HDAs, compared with quantitative PIMA® CD4 testing which is the current gold standard performed by trained nurses. Second, we assessed if HDAs can correctly perform and interpret serum-CrAg and urine-LAM tests for CM and disseminated TB respectively, and to compare the test performance with existing published data. Finally, we estimated potential cost savings associated with task sharing in this setting.

## Methods

### Study setting and population

Two HDAs were recruited for this study. They were trained and certified in HIV testing but had no prior experience with the VISITECT® CD4 LFA, urine LAM, or serum CrAg testing. The HDA is a novel HIV testing cadre in Malawi, with early findings showing feasibility and effectiveness in delivering HIV testing services [11]. The HDAs received a half-day training in advanced HIV disease, TB, and cryptococcal meningitis followed by a 5-day practical training in the laboratory. HDAs were also oriented to conduct tests using the WHO-prequalified VISITECT® CD4 rapid assay, consistent with international standards [12].

This Cross-sectional study was conducted at the Lighthouse ART Clinic at Kamuzu Central Hospital in Lilongwe, Malawi, between 18 August 2023 and 8 March 2024. Lighthouse ART clinic is a large cohort and well-established Centre of Excellence for comprehensive and integrated HIV management with over 12,700 clients alive on antiretrovirals and has played a central role in the country’s HIV response for over 15 years [13]. The clinic also serves as a referral site for complex HIV cases and primarily provides care to PLHIV, with few HIV-negative patients accessing Hepatitis B care. The daily clinic attendance averages 150 clients, with two new ART initiations per day and an average of 8 clients eligible for AHD screening among the new initiates, those presenting with high viral loads, clients re-engaging in care following default, very ill referrals from primary-level clinics, and children under 5 years of age. Nurses are responsible for sample collection for viral load monitoring, chemistry, hematology and other tests including LAM and CrAg tests which are sent to the on-site laboratory for further processing by laboratory technicians. In addition to sample collection, nurses run CD4 tests on PIMA machines that are placed within the blood draw area. Patients presenting with AHD are managed according to the Malawi National guidelines, which recommend serum CrAg and urine LAM testing for individuals with CD4 ≤ 200 cells/µL [14].

### Tests used

The VISITECT® CD4 test product (Omega Diagnostics, United Kingdom) was used as the semi-quantitative lateral flow assay [12,15]. The PIMA® CD4 test (Abbott, United States), a quantitative point-of-care device, was used as the reference assay throughout the study. Serum CrAg tests were performed using the CrAg® lateral flow assay (IMMY, United States), and urine LAM testing was conducted using the Determine™ TB LAM Ag assay (Abbott, United States). All assays were WHO-prequalified or WHO-recommended and were performed according to the manufacturers’ instructions and local standard operating procedures. For urine LAM testing, results were recorded as positive (band as dark or darker than the reference card line) or negative (no band or a band lighter than the reference card line). Test grading was not recorded.

### Eligibility criteria

All people living with HIV aged ≥18 years who were either initiating ART or referred for CD4 testing were eligible. Indications for CD4 testing included new HIV diagnosis, high viral load, reengagement in ART after treatment interruption of more than two months, or diagnosis of WHO stage 3 or 4 clinical conditions. Sequential individuals requiring CD4 testing were approached to provide written informed consent. Exclusion criteria included age < 18 years and being clinically unstable and unsuitable for outpatient care.

### Study design and procedures

After providing informed consent, participants were first seen by an HDA, who collected a finger-prick blood sample and immediately performed VISITECT® CD4 LFA testing in a separate consultation room. For participants with VISITECT® CD4 results ≤200 cells/mm³, HDAs collected a second capillary finger-prick sample to perform serum CrAg testing [16] and conducted urine LAM testing [17] using a self-collected urine specimen. All results were interpreted and recorded by the HDA on a standardized study form.

Participants were then referred to the on-site laboratory, where a nurse collected a 5-mL venous blood sample in an EDTA tube. The sample was split into two aliquots: one was used by the nurse to perform quantitative PIMA® CD4 testing as part of routine standard of care, and the second was used by a laboratory technician to perform VISITECT® CD4 LFA testing. For participants with PIMA® CD4 results ≤200 cells/mm³, nurses also performed serum CrAg testing and urine LAM testing as part of routine care.

VISITECT® CD4 LFA testing performed by laboratory technicians used venous EDTA blood. During routine laboratory workflow, some EDTA samples were stored briefly under refrigerated conditions prior to testing. All individual test results were recorded on case report forms, placed in sealed envelopes, and collected by a data clerk. HDAs, nurses, and laboratory technicians were blinded to each other’s test results. Only results from PIMA® CD4 testing, serum CrAg, and urine LAM performed by nurses as part of standard of care were made available to clinicians for patient management.

### Data analysis

We calculated the sensitivity, specificity, positive predictive value (PPV), and negative predictive value (NPV) of the VISITECT® CD4 lateral flow assay (LFA), using paired quantitative CD4 measurements obtained from the PIMA CD4 device (Abbott, USA) as the reference standard. The primary analysis assumed the PIMA CD4 measurement to be an accurate reference for classification of advanced HIV disease using the ≤ 200 cells/mm³ threshold.

However, PIMA CD4 measurements are known to exhibit analytical variability, particularly at lower CD4 counts, with reported variation of up to 15%, corresponding to approximately ±30 cells/mm³ at CD4 values below 200 cells/mm³. To account for potential misclassification arising from this measurement variability, we conducted a sensitivity analysis under a best-case reclassification assumption, in which VISITECT® CD4 LFA results were considered correctly classified when the corresponding PIMA CD4 result fell within the range of 170–230 cells/mm³ around the decision threshold.

To further explore potential bias due to imperfect reference standards, we applied a correction using the Gart and Buck method, assuming conditional independence between the index test (VISITECT® CD4 LFA) and the reference standard (PIMA® CD4). Reference standard sensitivity and specificity values for the PIMA® CD4 device were derived from published evaluations.

Predictive values depend on disease prevalence; therefore, PPV and NPV were estimated at two prevalence levels: the observed study prevalence (40%) and the estimated national prevalence for Malawi (28%). Sensitivity and specificity estimates are independent of prevalence and are therefore reported without adjustment. All analyses were conducted using standard diagnostic accuracy methods. Confidence intervals for sensitivity, specificity, PPV, and NPV were calculated using exact binomial methods.

A minimum sample of 308 paired patient samples was determined based on the ability to detect high inter-rater agreement (κ ≥ 0.90), assuming that approximately 45% of participants would have a CD4 count ≤200 cells/mm^3^, as previously observed at lighthouse [18]. This sample size also provided enough participants with and without advanced HIV disease to permit estimation of diagnostic accuracy (sensitivity and specificity) with acceptable precision.

For the costs, we compared the incremental cost per test of the different test and staffing modalities (PIMA vs. VISITECT® CD4 LFA; diagnostic assistant vs. nurse) in 2023 US dollars. Costs of consumables were based on unit prices, including procurement and shipping charges (Ministry of Health logistics data). Staff cost was estimated using the national wages at entry level of Malawi MOH salary scale and the average duration of the testing procedures. We did not include fixed device costs, quality assurance/ quality control costs or training costs.

### Ethical approval

The study protocol together with informed consent forms and data collection tools were approved by the National Health Sciences Research Committee (NHSRC; Reference Number: 2881). Written informed consent was obtained from all participants. Participants who were illiterate provided a thumb print on the form to indicate their consent to participate.

## Results

Between 18 August 2023 and 8 March 2024, we enrolled 308 participants. The median age was 39 years [interquartile range (IQR): 31–48], and 167 participants (54.5%) were female. The median CD4 count measured by the PIMA® device was 248 cells/mm³ [IQR: 136–483]. A total of 115 participants (37.3%) had CD4 counts ≤200 cells/mm³.

### Performance of VISITECT® CD4 LFA tests compared to PIMA CD4 test

**Fig 1:**
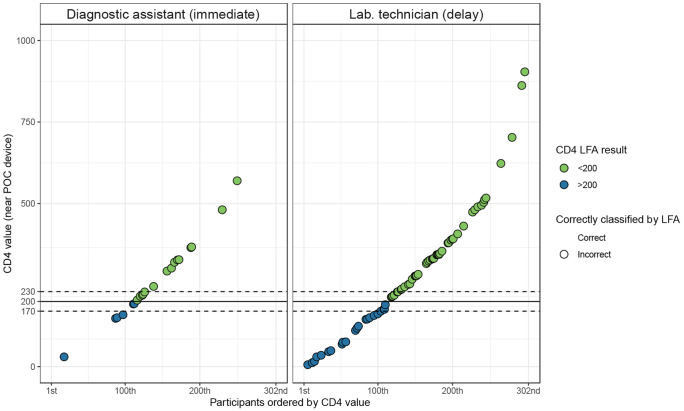
CD4 test results by PIMA® CD4 and VISITECT® CD4 lateral flow assay. CD4 cell counts measured using the quantitative PIMA® CD4 device performed by nurses (y-axis) and corresponding semi-quantitative VISITECT® CD4 LFA results performed by HIV diagnostic assistants (colour-coded points). Horizontal lines indicate CD4 cell count thresholds. Light-shaded points indicate concordant results, while dark-shaded points indicate discordant results. Results for six participants with CD4 counts >1000 cells/mm³ (all concordant) are not shown. **B:** Sensitivity and specificity estimates using the Gart and Buck correction for imperfect reference standards, assuming conditional independence between the reference standard and index test. Accuracy of the reference standard based on Wade et al. (2015).

CD4 testing was performed on all 308 participants by both HIV diagnostic assistants (HDAs) using the VISITECT® CD4 lateral flow assay and nurses using the PIMA® CD4 device. One participant did not obtain a VISITECT® CD4 LFA result from the laboratory technician (Fig 1).

The diagnostic accuracy of the VISITECT CD4 LFA varied notably by sample type and testing cadre with HDAs reading more accurately on fresh capillary blood compared to laboratory technicians using EDTA samples.

When performed by HDAs on fresh capillary blood, the test demonstrated high sensitivity (95%; 95% CI: 89–98) and specificity (92%; 95% CI: 88–96), indicating excellent ability to correctly identify individuals with and without advanced HIV disease. The positive predictive value (PPV) was 88%, suggesting that most individuals classified as having CD4 < 200 cells/mm³ were true positives, while the negative predictive value (NPV) was 97%, reflecting a very low likelihood of missed advanced disease. Only a small number of false negatives (6 cases) were observed, reinforcing the test’s reliability for ruling out advanced HIV disease in this setting.

In contrast, performance declined when the assay was conducted by laboratory technicians on EDTA samples. Sensitivity was observed at 81% (95% CI: 72–88) and specificity at 76% (95% CI: 69–81. The PPV was 66%, reflecting a higher proportion of false positives, while the NPV was 87%, as shown in Table 1.

**Table 1:** Diagnostic accuracy VISITECT® CD4 LFA compared to PIMA CD4.

Index test	PIMA CD4 test (reference standard)		
	Reference test <200 cells/mm^3^	Reference test >200 cells/mm^3^	
VISITECT® CD4 LFA test on fresh capillary blood by HDAs.			
Index test <200 cells/mm^3^	109	15	PPV: 0.88 (0.81–9.93)
Index test >200 cells/mm^3^	6	178	NPV: 0.97 (0.93–0.99)
	Sensitivity: 0.95 (0.89–0.98)	Specificity: 0.92 (0.88–0.96)	
VISITECT® CD4 LFA test on EDTA samples by Laboratory technicians			
Index test <200 cells/mm^3^	93	47	PPV: 0.66 (0.58–0.74)
Index test >200 cells/mm^3^	22	145	NPV: 0.87 (0.81–0.92)
	Sensitivity: 0.81 (0.72–0.88)	Specificity: 0.76 (0.69–0.81)	

**Legend:** PPV: Positive predictive value, NPV: negative predictive value (at observed prevalence)

To assess the generalizability of predictive values to routine programmatic settings, we estimated positive and negative predictive values using a population prevalence of advanced HIV disease of 28%, corresponding to national estimates for Malawi. At this prevalence, the negative predictive value and positive predictive value for VISITECT® CD4 LFA performed by HIV diagnostic assistants were 0.83 (95% CI: 0.74–0.89) and 0.98 (95% CI: 0.95–0.99), respectively. S1 Table presents the results of a sensitivity analysis using a ‘best-case scenario’, in which all VISITECT® CD4 LFA results with corresponding PIMA® CD4 values within 15% of the decision threshold were considered correctly classified.

### Results of serum-CrAg and urine-LAM

Of the 115 individuals with VISITECT® CD4 LFA CD4 < 200 cells/mm³, 107 underwent CrAg testing by both the nurse and the HDA, while 105 underwent LAM testing by both cadres. Two individuals were missing CrAg test results, and three were missing LAM test results from the nurses. Additionally, five individuals were found to have CD4 counts >200 cells/mm³ based on Visitect testing conducted by the HDAs and were therefore deemed ineligible for CrAg and LAM testing. Among the 193 participants with CD4 counts >200 cells/mm³ measured using PIMA CD4 devices, five had serum CrAg tests and seven had urine LAM tests performed by nurses.

Among the 107 individuals who had serum CrAg testing performed by both an HDA and a nurse, 5 tested CrAg positive of whom 3 were also identified as positive by the HDAs. There was 100% concordance for negative results. Overall agreement was 98% (Cohen’s kappa = 0.75).

In 105 participants where urine LAM was performed by both HDAs and nurses, 97 had concordant negative test result, six concordant positive results whilst two samples that tested positive by nurses were recorded as negative by HDAs. Overall agreement was 98% (Cohen’s kappa = 0.85). There was one additional case where the nurse obtained a positive LAM result, but the HDA did not perform the test, and there were three cases where the HDA obtained positive LAM results, but the nurse did not perform the test as shown in table 2.

**Table 2:** Comparison of urine LAM and serum CrAg testing.

	Serum CrAg testing by HIV Diagnostic Assistance		
Serum CrAg testing by Nurses	CrAg positive	CrAg negative	Total
CrAg positive	3	2	5
CrAg negative	0	102	102
Total	3	104	107
	Urine LAM testing by HIV Diagnostic Assistance		
Urine LAM testing by Nurses	LAM positive	LAM negative	Total
LAM positive	6	2	8
LAM negative	0	97	97
Total	6	99	105

### Cost

We estimated that per-test costs of consumables to Malawi HIV programme (using low-income country preferential pricing) in 2024 US dollars were $7.78 for the PIMA CD4PIMA test and $4.17 for VISITECT® CD4 LFA. The PIMA CD4 took 35 minutes of staff time (including preparation, blood draw and documentation) and the VISITECT® LFA took 60 minutes. The cost for 35 minutes of nurse staff time was $0.91 and for 60 minutes of diagnostic assistant time was $1.07. Overall incremental cost per CD4 test was $8.69 for PIMA CD4 quantitative test by a nurse and $5.24 for semi-quantitative VISITECT® LFA by a diagnostic assistant.

## Discussion

The WHO advanced HIV disease guidelines suggested programs consider task sharing for performing the advanced HIV point-of-care diagnostics although an evidence gap was noted, particularly with more complex assays. Our results show good performance of VISITECT®CD4 LFA in the hands of lay personnel with only a few days of targeted training and it was feasible to task shift to HDAs. In contrast, lower diagnostic performance was observed when VISITECT® CD4 LFA was conducted by laboratory technicians on EDTA samples, noting that testing occurred within routine workflows and included a mix of immediately processed samples and some stored for several hours at refrigerator temperature.

Overall, the performance characteristics for VISITECT®CD4 by HDAs on fresh capillary blood was consistent with results from previously published reports. Seven studies allowing individual patient data extraction on VISITECT® CD4 LFA test performance were identified in the literature [19–23]; the diagnostic performance in our study was at the upper range of sensitivity (62^20^–97^18^%) and specificity (71^24^–95^18^%) of previous evaluations. We attribute the poor performance of VISITECT LFAs on stored blood to being most likely related to pre-analytic factors such as sample degradation over time rather than poor interpretation by lab technicians. We urge those implementing VISITECT® LFA role out to consider pre-analytic factors to optimize test accuracy.

We are confident that using HDAs to perform CD4 and other advanced HIV disease point of care diagnostic tests is feasible without reducing quality compared to qualified nurses. This finding is consistent with evidence from Uganda, where successful decentralization of CD4 testing using the VISITECT CD4 LFA demonstrated high sensitivity (100%) and moderate specificity (81%), with variability across clinic settings. Importantly, while specificity was lower, misclassified cases tended to have CD4 counts well above the 200 cells/µL threshold, suggesting limited clinical risk of missed advanced HIV disease. These findings, together with our results, reinforce that simplified, point-of-care CD4 testing approaches can be effectively implemented outside traditional laboratory settings, even when conducted by lay cadres [24]. Task-sharing has played a critical role in Malawi’s HIV testing program over the years by enabling far more PLHIV to be diagnosed and started on ART than would have otherwise been possible. The same strategy can now be used to accelerate the scale-up of advanced HIV disease services.

Whilst we have shown that HDAs with minimal training were able to use the VISITECT® CD4 LFA test, the current Visitect lateral flow test takes longer than other device-based tests (approximately 40–45 minutes compared to 30 minutes) and has more complicated steps such as adding buffer at specific time points. These time-sensitive steps make the test less suitable for “batch” testing (i.e., one person running multiple tests at the same time) and for completing other tasks while waiting for the next step in the VISITECT® LFA test. There is also limited experience and guidance on quality assurance and quality control for the VISITECT® LFA, compared to PIMA CD4 where QC cartridges and user-friendly instructions for use are readily available. Our findings suggest that operational factors, including delays between sample collection and result generation, may influence VISITECT® CD4 LFA performance, reinforcing the role of ongoing training and support for testing personnel as part of quality assurance measures.

Serum-CrAg and the urine-LAM results showed a high concordance between the results of nurses and HDAs, but the low number of positive samples limited the precision of the estimates for diagnostic accuracy. Our results are consistent with previously published reports from Lesotho, showing that CrAg LFA screening by lay cadres is feasible [25]. Field implementation evaluations in rural Malawi also suggest that trained HDAs can perform and interpret rapid tests for opportunistic infections and refer presumed AHD patients to clinicians [26]. There was less concordance in urine LAM readings than serum CrAg readings – presumably because reading a urine LAM test can be subjective when it comes to comparing faint lines with the reference card.

Our cost estimates indicate that using the VISITECT® CD4 LFA test in this setting could result in up to 30% incremental cost savings, with an additional 13% savings achievable by task sharing testing to HDAs. Similar cost and implementation considerations have been reported in other programmatic evaluations of the VISITECT® CD4 test. A pragmatic multisite study in Uganda found that VISITECT® CD4 had lower consumable costs than device-based CD4 platforms, with overall cost advantages dependent on staff cadre, clinic workflow, and testing volume [19].

In addition to these cost savings, the VISITECT®CD4 LFA facilitates the expansion of advanced HIV diagnostic services to primary-level care health facilities. Broader policy and implementation analyses have also identified CD4 lateral flow assays, including VISITECT® CD4, as cost-advantageous options for advanced HIV disease screening in decentralized and resource-limited settings, particularly where laboratory infrastructure and reliable electricity are limited [27].

This is particularly relevant in a decentralized ART program such as Malawi’s [13], where approximately 835 treatment sites provide ART nationwide. In many of these sites, electricity is unreliable or unavailable, healthcare staff are limited, and laboratory technicians are absent. Under these conditions, it is unlikely that nurses, who generally manage these smaller ART clinics, would be able to perform the VISITECT® CD4 LFA test due to competing responsibilities.

HDAs were introduced in the Malawian HIV program to task-share HIV testing using simple algorithms at the point of care [9,11]. It is therefore sensible to consider using this lay cadre for additional advanced HIV disease (AHD) screening tests. However, because these tests must meet specific quality standards, existing policies on lay provider testing may need to be adapted to strengthen quality control measures. The VISITECT® CD4 LFA test may be less suitable for busy HIV referral clinics with high patient volumes due to the long incubation period and the time-sensitive repeated application of buffer. Delivering a single-day “one stop shop” care to multiple patients in parallel is more challenging with the VISITECT® CD4 LFA, which requires approximately 45 hands-on minutes, compared with 20 hands-on minutes for PIMA CD4 testing.

In clinical settings with better-trained staff and potential inpatient care [28], knowing CD4 counts below 100 cells/mm³ or even below 50 cells/mm³ can inform differential diagnoses and guide therapeutic decisions. In contrast, deploying lay cadres in smaller peripheral facilities with fewer patients, where skilled health workers are occupied with clinical duties, appears to be more feasible. In such settings, patients can be screened and receive same-day treatment for any AHD condition identified. This approach may also be advantageous in sites with unreliable electricity, which limits the use of traditional CD4 machines [29].

A limitation of our study is that the HDAs were full-time and focused solely on AHD testing; they did not perform other tests such as HIV, syphilis, or hepatitis B screening as would typically occur in most health facilities. This dedicated focus may have contributed to their excellent performance. While the sample size was sufficient for estimating diagnostic performance, the number of participants particularly those with LAM or CrAG positive results, was limited, which may have reduced the precision of sensitivity, specificity, and agreement estimates. Additionally, a small number of urine LAM results were not paired between HDAs and nurses, which may have affected the estimation of agreement.

## Conclusion

Lay providers, with brief training, can deliver advanced HIV testing services using LFAs for CD4, urine-LAM, and serum CrAg with accuracy comparable to trained nurses. These findings support the decentralized implementation of advanced HIV disease testing by lay providers, particularly in rural clinics that see few patients per day, lack other CD4 testing devices, or have unreliable electricity and limited laboratory facilities. This implementation model appears highly feasible, potentially cost-saving, and could play a critical role in expanding access to advanced HIV disease testing and treatment, with significant population-level impact.

## Supporting information

S1 Table Sensitivity estimates for CD4 testing. (A) Best-case scenario, in which all quantitative tests within ±15% of the threshold are considered correctly classified. Sensitivity, specificity, positive predictive value (PPV), and negative predictive value (NPV) are shown for: (i) VISITECT® CD4 LFA testing on fresh capillary blood by HIV diagnostic assistants and (ii) VISITECT® CD4 LFA testing on EDTA samples by laboratory technicians. Reference standard thresholds were <230 cells/mm³ if LFA < 200 cells/mm³ and >170 cells/mm³ if LFA > 200 cells/mm³. (B) Sensitivity and specificity estimates using the Gart and Buck correction for imperfect reference standards, assuming conditional independence between the reference standard and index test. Accuracy of the reference standard based on Wade et al. (2015).
(DOCX)

S1 Fig Reclassification of points under the best-case scenario. The top panel shows all results and their classification. The bottom left panel shows results in the range of 120–180 CD4 cells/mm³ classified using original values, assuming no measurement error in the reference standard. Tests in which the VISITECT® CD4 LFA result was < 200 cells/mm³ and the PIMA® CD4 result was > 200 cells/mm³ were classified as false positives (orange). The bottom right panel shows classification under the best-case scenario, in which four results with VISITECT® CD4 LFA < 200 cells/mm³ and PIMA® CD4 values between 200 and 230 cells/mm³ were reclassified as true positives (yellow). Similarly, two results with VISITECT® CD4 LFA > 200 cells/mm³ and PIMA® CD4 values between 170 and 200 cells/mm³ were reclassified as true negatives (light blue).
(TIF)

S2 Fig Estimated cost per CD4 test result. Estimated consumable costs and Ministry of Health entry-level wage assumptions for different testing cadres in Malawi, expressed in 2024 US dollars.
(TIF)

S1 Text Literature search for previously published VISITECT® CD4 test results including search strategy, study selection criteria, data extraction, and summary of diagnostic performance (sensitivity and specificity) across identified studies. (DOCX)

S3 Fig Forest plot of published sensitivity and specificity estimates of VISITECT® CD4 tests in comparison with results from the Lighthouse study. Blood specimen types used in each study are indicated. Between-study heterogeneity was high for sensitivity (χ² = 54, df = 10, p < 0.001, I² = 92%) and specificity (χ² = 79, df = 10, p < 0.001, I² = 91%).
(TIF)

S1 Data Individual-level dataset used for analysis. Contains VISITECT® CD4 LFA results, PIMA® CD4 reference values, and CrAg and LAM test results performed by HIV diagnostic assistants and nurses.
(XLSX)
