# Supplementary material for: High performance of point-of-care rapid tests for advanced HIV disease diagnosis by lay providers in Malawi: Results from a prospective diagnostic accuracy study supporting decentralized advanced HIV disease screening
Source: PLoS One. 2026 Jun 18;21(6):e0340955. doi: 10.1371/journal.pone.0340955 (PMC13278447; doi:10.1371/journal.pone.0340955)
Supplement: S1 Table — (A) Best-case scenario, in which all quantitative tests within ±15% of the threshold are considered correctly classified. Sensitivity, specificity, positive predictive value (PPV), and negative predictive value (NPV) are shown for: (i) VISITECT® CD4 LFA testing on fresh capillary blood by HIV diagnostic assistants and (ii) VISITECT® CD4 LFA testing on EDTA samples by laboratory technicians. Reference standard thresholds were <230 cells/mm³ if LFA < 200 cells/mm³ and >170 cells/mm³ if LFA > 200 cells/mm³. (B) Sensitivity and specificity estimates using the Gart and Buck correction for imperfect reference standards, assuming conditional independence between the reference standard and index test. Accuracy of the reference standard based on Wade et al. (2015). (DOCX) [file pone.0340955.s001.docx]

**Supplementary table 1:** Sensitivity estimates for CD4 testing

**A:** Best case scenario – all quantitative tests within +/- 15% of threshold are considered correctly classified.

| Index test | PIMA® CD4 device test (reference standard) | |  |
| --- | --- | --- | --- |
|  | Reference test <230 cells/mm^3^ if LFA is <200 cells/mm^3^  **or**  Reference test >170 cells/mm^3^ if LFA is >200 cells/mm^3^ | Reference test <230 cells/mm^3^ if LFA is <200 cells/mm^3^  **or**  Reference test >170 cells/mm^3^ if LFA is >200 cells/mm^3^ |  |
| LFA test on fresh capillary blood by HIV diagnostic assistants. |  |  |  |
| Index test <200 cells/mm^3^ | 114 | 10 | PPV: 0.90 (0.84-0.95) |
| Index test >200 cells/mm^3^ | 4 | 180 | NPV: 0.98 (0.95 – 0.99) |
|  | Sensitivity 0.97 (0.91 – 0.99) | Specificity 0.94 (0.89 – 0.97) |  |
|  |  |  |  |
| LFA test on EDTA samples by laboratory technicians |  |  |  |
| Index test <200 cells/mm^3^ | 98 | 31 | PPV: 0.71 (0.62-0.78) |
| Index test >200 cells/mm^3^ | 18 | 148 | NPV: 0.89 (0.83 – 0.93) |
|  | Sensitivity: 0.84 (0.77-0.91) | Specificity: 0.78 (0.72 – 0.84) |  |

|  | PIMA® CD4 device test (reference standard) | |  |
| --- | --- | --- | --- |
| LFA test on fresh capillary blood | Reference test <230 cells/mm^3^ if LFA is <200 cells/mm^3^  **or**  Reference test >170 cells/mm^3^ if LFA is >200 cells/mm^3^ | Reference test <230 cells/mm^3^ if LFA is <200 cells/mm^3^  **or**  Reference test >170 cells/mm^3^ if LFA is >200 cells/mm^3^ |  |
| Index test <200 cells/mm3 | 114 | 10 | PPV: 0.90 (0.84-0.95) |
| Index test >200 cells/mm3 | 4 | 180 | NPV: 0.98 (0.95 – 0.99) |
|  | Sensitivity 0.97 (0.91 – 0.99) | Specificity 0.94 (0.89 – 0.97) |  |

**B:** Using Gart and Buck correction for imperfect reference standard (NB. assumes conditional independence of reference standard and index test). Accuracy of reference standard from Wade et. al. (https://pmc.ncbi.nlm.nih.gov/articles/PMC4149645/pdf/qai-66-e98.pdf)

| Sensitivity: 0.95 (0.83 – 0.98) |
| --- |
| Specificity 0.98 (0.94 – 0.99) |
