# Supplementary figures and images for: High performance of point-of-care rapid tests for advanced HIV disease diagnosis by lay providers in Malawi: Results from a prospective diagnostic accuracy study supporting decentralized advanced HIV disease screening
Source: PLoS One. 2026 Jun 18;21(6):e0340955. doi: 10.1371/journal.pone.0340955 (PMC13278447; doi:10.1371/journal.pone.0340955)

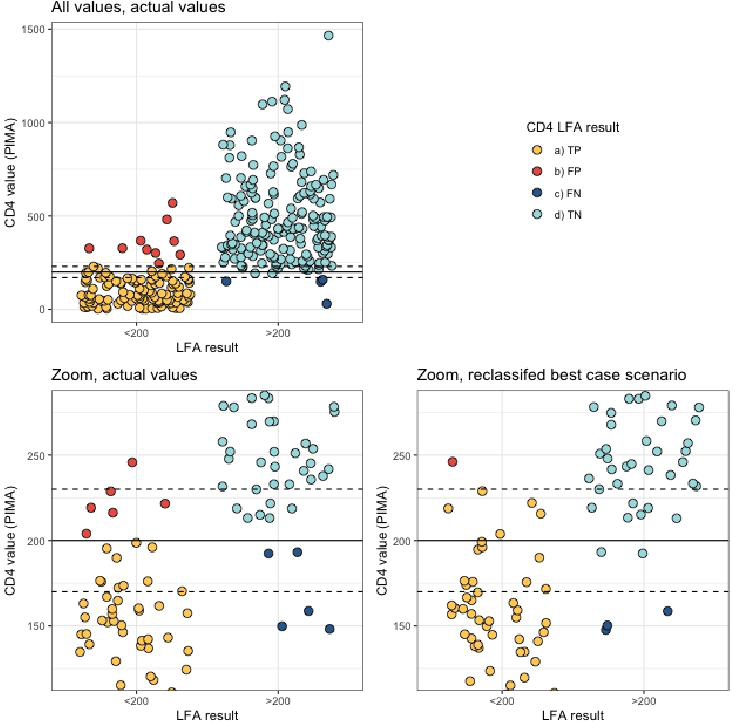

Supplement: S1 Fig — The top panel shows all results and their classification. The bottom left panel shows results in the range of 120–180 CD4 cells/mm³ classified using original values, assuming no measurement error in the reference standard. Tests in which the VISITECT® CD4 LFA result was < 200 cells/mm³ and the PIMA® CD4 result was > 200 cells/mm³ were classified as false positives (orange). The bottom right panel shows classification under the best-case scenario, in which four results with VISITECT® CD4 LFA < 200 cells/mm³ and PIMA® CD4 values between 200 and 230 cells/mm³ were reclassified as true positives (yellow). Similarly, two results with VISITECT® CD4 LFA > 200 cells/mm³ and PIMA® CD4 values between 170 and 200 cells/mm³ were reclassified as true negatives (light blue). (TIF) [file pone.0340955.s002.tif]

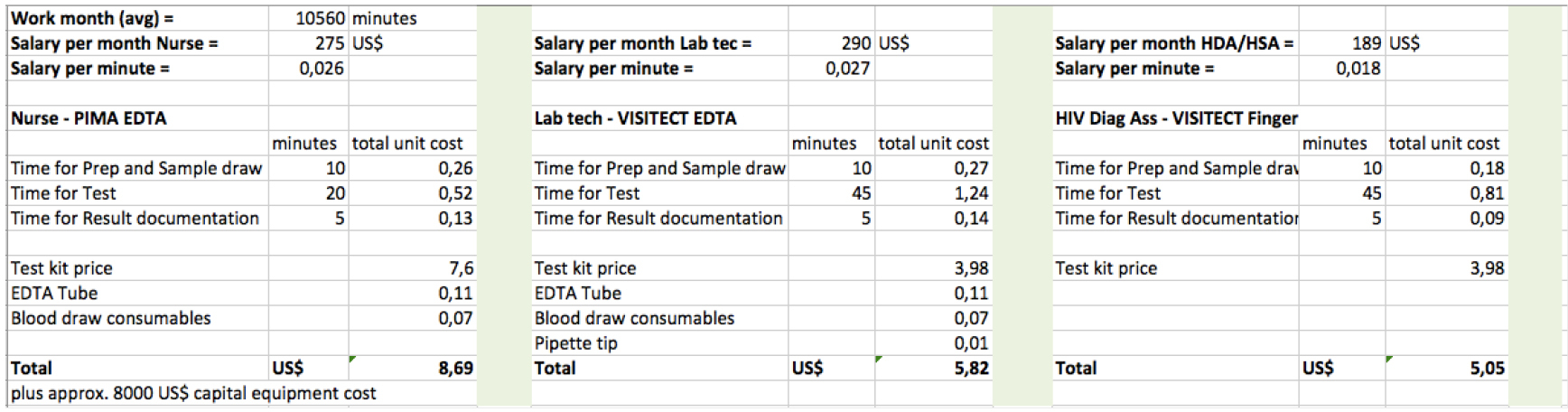

Supplement: S2 Fig — Estimated consumable costs and Ministry of Health entry-level wage assumptions for different testing cadres in Malawi, expressed in 2024 US dollars. (TIF) [file pone.0340955.s003.tif]

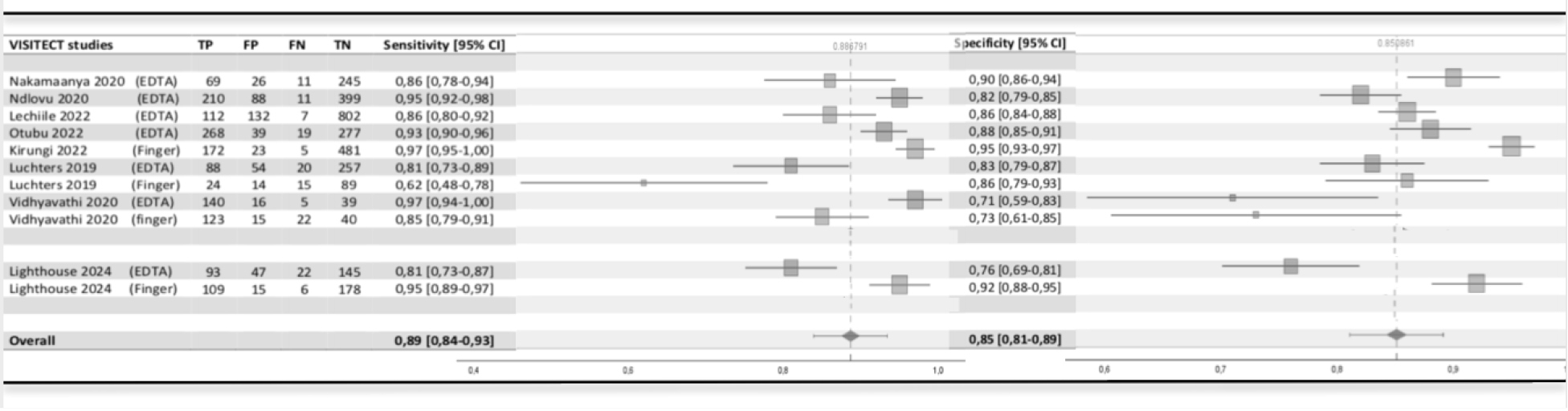

Supplement: S3 Fig — Blood specimen types used in each study are indicated. Between-study heterogeneity was high for sensitivity (χ² = 54, df = 10, p < 0.001, I² = 92%) and specificity (χ² = 79, df = 10, p < 0.001, I² = 91%). (TIF) [file pone.0340955.s005.tif]
