# Supplementary material for: High performance of point-of-care rapid tests for advanced HIV disease diagnosis by lay providers in Malawi: Results from a prospective diagnostic accuracy study supporting decentralized advanced HIV disease screening
Source: PLoS One. 2026 Jun 18;21(6):e0340955. doi: 10.1371/journal.pone.0340955 (PMC13278447; doi:10.1371/journal.pone.0340955)
Supplement: S1 Text — (DOCX) [file pone.0340955.s004.docx]

**Literature search for previously published VISITECT® CD4 test results**

**Search criteria.**

Literature was searched in PubMed and Google Scholar using the search string “CD4” AND “point‑of‑care” AND (“lateral flow assay” OR “VISITECT”) to identify studies assessing test characteristics of the VISITECT® CD4 test. Subsequently, a targeted Google Scholar search using the same terms was performed to identify relevant conference poster presentations not indexed in the above‑mentioned databases. All studies that allowed extraction of individual patient data from the published report or poster were included, summarised, and analysed using a random‑effects model. Studies using the VISITECT® CD4 350 test were included, as it functions on the same test principle.

**Comparison of study results with previously reported test performance.**

Seven studies allowing individual patient data extraction on VISITECT® CD4 test performance were identified in the literature [10,19–24, 29]. Two studies [20,24] reported disaggregated results for different sample types (EDTA versus finger‑prick whole blood), and data were analysed accordingly. All published studies were conducted in laboratory settings except those by Otubu et al. [23] and Kirungi et al. [29], where VISITECT® CD4 testing was performed by facility‑based health care workers, and Luchters et al. [21], in which finger‑prick VISITECT® CD4 testing was performed by nurses. The results are summarised in S3 Fig. Sensitivity ranged from approximately 62% to 97%, while specificity ranged from approximately 71% to 95%.

Despite substantial heterogeneity, pooled estimates across all studies, including data from the present study, showed an overall sensitivity of 89% (95% CI: 84–93%) and an overall specificity of 85% (95% CI: 81–89%).
