# Supplementary material for: High performance of point-of-care rapid tests for advanced HIV disease diagnosis by lay providers in Malawi: Results from a prospective diagnostic accuracy study supporting decentralized advanced HIV disease screening
Source: PLoS One. 2026 Jun 18;21(6):e0340955. doi: 10.1371/journal.pone.0340955 (PMC13278447; doi:10.1371/journal.pone.0340955)
Supplement: Attachment — Submitted filename: Response to Reviewers.docx [file pone.0340955.s008.docx]

**Response to Reviewers**

**Manuscript ID: PONE-D-25-68943**
**Title: High performance of point-of-care rapid tests for advanced HIV disease diagnosis by lay providers in Malawi: Results from a prospective diagnostic accuracy study supporting decentralized advanced HIV disease screening**

Dear Academic Editor,

We thank you and the reviewers for the careful evaluation of our manuscript and for the constructive and insightful comments provided. We have revised the manuscript extensively in response to all points raised.

To facilitate review of the revisions, we provide a detailed, point‑by‑point response in the accompanying table, in which each reviewer comment is reproduced verbatim alongside our corresponding response and a description of the changes made in the manuscript. All revisions are reflected in the revised manuscript.

In summary, the key revisions include:

- Clarification of the diagnostic assays evaluated, with explicit identification of the VISITECT® CD4 lateral flow assay in the Abstract, Introduction, and Methods;
- Inclusion of relevant prior literature on VISITECT® CD4 performance and clearer articulation of the novelty and added value of this study;
- Improved methodological clarity regarding specimen types, handling, and reference standards;
- Enhanced presentation and interpretation of diagnostic accuracy results, including justification of prevalence assumptions used for predictive value estimation;
- Reorganization and refinement of the Results and Discussion sections to remove redundancy, strengthen contextualization, and improve readability;
- Comprehensive correction of grammatical, typographical, and formatting inconsistencies across the manuscript.

We believe that these revisions have substantially strengthened the manuscript and addressed all concerns raised by the reviewers. We appreciate the opportunity to revise the work and thank you again for your time and consideration.

Sincerely,

Agness Thawani
The Corresponding Author

| **Reviewer** | **Comment** | **Feedback** |
| --- | --- | --- |
|  | The authors are attempting to address an important subject but they are several weaknesses with this report : |  |
| **Reviewer 1** | There are several gramma/ typographical errors that could have been taken care of before submission | The manuscript has been carefully revised, and grammatical and typographical errors have been corrected throughout. |
|  | The name of the LFA being evaluated is only disclosed much later in the methodology section and is a bit surprising when it is finally mentioned as Visitect, yet through out the abstract or the introduction, there is no reference to visitect. | Thank you for this comment. We have revised the abstract to explicitly identify the VISITECT® CD4 lateral flow assay at first mention, ensuring that the diagnostic test under evaluation is clearly stated upfront rather than only appearing later in the Methods section. |
|  | There are some publications on visitect that should have been referenced, the authors then needed to make the case for what is different in the work that is being presented from previous evaluations. | We have revised the Introduction to reference prior VISITECT® CD4 evaluations and to clarify the added value of this study, which is the assessment of diagnostic accuracy, feasibility, and cost of VISITECT® CD4 testing when performed by lay carder. |
|  | Abstract: There is ambiguity. The name of the tool being evaluated is not mentioned, | We revised the abstract to explicitly specify the type of lateral flow assays evaluated for CD4 testing |
|  | In the abstract results section, one then realised that this evaluation extends beyond CD4 LFA. | We have revised the abstract to clarify upfront that the evaluation includes not only CD4 lateral flow assay testing (VISITECT® CD4) but also serum cryptococcal antigen and urine lipoarabinomannan diagnostics, ensuring the full scope of the evaluation is clear from the outset. |
|  | The authors should consider revising the title to take care of the additional tests | We revised the title to reflect the evaluation of advanced HIV disease diagnostics, which include CD4 testing as the defining entry-point assay, as well as reflex serum cryptococcal antigen and urine TB-LAM testing. The revised title accurately reflects the scope of diagnostics evaluated without overstating test-specific accuracy claims. |
|  | Line 68 : Large proportion is ambiguous, gives impression more PLHIV are AHD than are controlled, yet this is not the case? Needs revision. | We agree that the phrase “large proportion” was ambiguous. We have revised the text to quantify the burden more precisely, stating that approximately one quarter to one third of people living with HIV presenting to care have advanced HIV disease, thereby avoiding any implication that the majority of PLHIV have AHD. |
|  | Line 74, can abbreviate PLHIV here | We have introduced the abbreviation in the first sentence and now use “PLHIV” consistently after its first definition. |
|  | Line 79-81 should be referenced | We added appropriate references to support the role of CD4 testing in identifying advanced HIV disease and the associated risk of opportunistic infections. |
|  | Line 93, does this test have a name at all or it is just CD4 LFA? | The test name has been included as indicated above |
|  | line 97- second objective comes as a bit of a surprise, the title is silent, consider revising the title a | The Title has been revised accordingly to include all tests assessed |
|  | line 126, what quantitative NPOC device is this? It is important to name it, so readers can reference | PIMA has been mentioned in the added text above |
|  | Line 133; Tests used: This needs to been mentioned earlier, until one gets to this line does one realise that the LFA being evaluated is visitect. See earlier comment on the same. Adn no reference to other works on visitect. There are several publications on visitect performance, some reference needs to be made to these in the introductory section, then what is this study has done differently to deserve being published Need to be clear on : 1. CD4 test LFA being evaluated? Name and manufacturer, has it been evaluated before? 2. Which test is the reference standard, is it PIMA semi quantitative or Visitect? 3. If CD4 LFA being evaluated is not visitect, clearly mention in the methodology that CD4 LFA is also being compared against visitect. | We have revised the Abstract and Introduction to explicitly identify the VISITECT® CD4 lateral flow assay (Omega Diagnostics) as the index test and to cite prior publications on its diagnostic performance and usability. We have also clarified in the Methods that quantitative PIMA® CD4 testing is the reference standard. The Introduction now highlights that the added value of this study is the evaluation of VISITECT® CD4 LFA performance, feasibility, and costs when implemented by lay providers in routine programmatic settings. |
|  | line158: Name the assay | We have revised the Methods section to explicitly name the assay as the VISITECT® CD4 lateral flow assay and to ensure consistent use of the assay name throughout the description of study procedures |
|  | Line 167, name the near point of care to remove ambiguity | Replaced term on NPOC with PIMA device |
|  | Line 169 to 174, needs to be clear if CrAg was performed on venous blood collected for NPOC, or finger stick blood was collected. What is sample type recommended by the manufacturer. For urine lam was a fresh sample collected? How long before testing ? | We have revised the Methods section to clearly specify the specimen types and timing of testing. Serum CrAg testing was performed using finger‑prick capillary blood, in line with the manufacturer’s instructions for use, which indicate that whole blood collected with potassium EDTA, sodium citrate, or sodium heparin anticoagulants is acceptable; the relevant reference has been added. Urine LAM testing was conducted using freshly collected urine specimens, which were tested immediately after collection. These clarifications have now been included in the manuscript. |
|  | Line 173 to 175- Why storage, why not test immediately. What is the sample type recommended for CD4 LFA? Refer to the SOP for visitect and clarify that using venous blood did not deviate from the recommended SOP. And is storage of samples the current practice? Initially, one gets the impression that comparison of CD4 LFA test by HDA, is comparing to qualified health staff using the exact methodology , then later one learns that different samples are used, and there is storage | We have revised the Methods section to clarify laboratory sample handling and workflow. According to the VISITECT® CD4 lateral flow assay standard operating procedures, both capillary finger‑prick blood and venous EDTA blood are validated specimen types, and use of venous EDTA blood therefore did not deviate from manufacturer recommendations. Venous EDTA blood was used for laboratory‑based VISITECT® CD4 testing to minimise repeated finger‑pricks and to allow parallel testing with PIMA® CD4 from the same blood draw.  Testing was performed within the manufacturer‑recommended time window for EDTA samples; storage was consistent with routine laboratory practice and is now clarified in the manuscript. We have also revised the text to clearly distinguish between point‑of‑care VISITECT® CD4 LFA testing by HDAs using finger‑prick blood and laboratory‑based VISITECT® CD4 LFA testing by technicians using venous EDTA blood, addressing any ambiguity regarding comparability of testing approaches. |
|  | 179 to 181; Clarity? which specific results | We have revised the text to explicitly specify that only nurse‑performed standard‑of‑care results (PIMA® CD4, urine LAM, and serum CrAg) were shared with clinicians for patient management. |
|  | Line 186 to 188, review whole sentence and revise as needed, it is difficult to read due to gramma/ typos | The sentence has been improved |
|  | Line 190. Clarify, two sensitivity analysis, but I only see one analysis below? | We agree that the wording in the original version was unclear. We have revised the manuscript to avoid this ambiguity. Specifically, we no longer refer to “two sensitivity analyses.” The revised text now clearly describes one sensitivity analysis, namely a best‑case reclassification analysis accounting for measurement variability of the PIMA CD4 reference standard. The additional analysis using the Gart and Buck method is now described as an exploratory correction for reference standard imperfection rather than a second sensitivity analysis. These revisions align the Methods, Results, and Supplementary Materials and clarify the analytical approach. |
|  | Line 199: does this mean the sample was not adequate for accuracy evaluation (sensitivity and Specificity), yet accuracy is the main objective for this evaluation and not reproducibility? | We have revised the wording to clarify that the sample size assumption regarding inter‑rater agreement was used to ensure an adequate distribution of participants across CD4 categories. This sample size was sufficient to support estimation of diagnostic accuracy measures (sensitivity and specificity), which remained the primary objective of the study. |
|  | Line 247- predictor or predictive, and why focus on 28% simulated prevalence , why not report actual findings from this study? | Thank you for noting this. We have corrected the typographical error and changed “predictor” to “predictive.” The observed study prevalence (40%) is reported in Table 1 and used for the primary PPV and NPV estimates. The additional estimates at 28% prevalence were included to illustrate how predictive values would vary at the national prevalence of advanced HIV disease in Malawi, thereby improving generalizability of the findings beyond the study setting. We have clarified this in the manuscript. |
|  | Line 321 is repetition. review and remedy as needed | We have removed the repetition |
|  | 403- Any comment on Sample size, was it adequate for accuracy evaluation? line 410, brief or minimal ? | The target sample size of 308 paired observations was chosen pragmatically to ensure sufficient precision for estimating diagnostic accuracy and agreement. With an expected advanced HIV disease prevalence of about 40 to 45 percent, this sample size was expected to include over 100 participants with CD4 ≤200 cells per mm³, allowing sensitivity and specificity to be estimated with acceptable precision. |
|  | References don't seem to follow the seem to follow the same format throughout. | We have reviewed and revised the reference |
| **Reviewer 2** | I commend team Malawi for working on this important piece of work which is relevant and adds to the evidence in favour of visitect use. The paper is well written and flows wells, the results and discussion can be improved, A few edits as highlighted below: |  |
|  | Please define the eligibility for one to be called a AHD. How are these selected by the ministry of health. Who qualifies?  To help give context to the results, please describe the patient burden at these HIV clinic where the study was run. Is it a low, medium or high volume clinic? Are general outpatients seen in the same clinic? Do the nurse run only CD4 tests and no other lab test? The bulk of work that has to be run affects the quality of result from our experience as busy sites are not able to be patient enough to obey the timelines for visitect testing affecting the overall result. You mention abit at the end of the paper but detail this in the study design section. | We have addressed this comment by explicitly defining advanced HIV disease in the Methods according to Malawi National Guidelines as CD4 ≤200 cells/µL, with eligibility determined through routine Ministry of Health criteria. We have also revised the study setting to describe the Lighthouse ART Clinic as a high‑volume referral site and to clarify patient load, case mix, and nursing workload, providing context for the testing conditions under which the study was conducted. |
|  | Consider using the type of machine used as the reference i.e PIMA instead of near POC device test as this might be more relatable to the readers. Near POC device might be a term used in some areas but not conventional. What makes it even more confusing is that both visitect and PIMA are point of care machines. | We agree and have revised the manuscript to consistently refer to the quantitative reference assay as the PIMA CD4 test, clearly distinguishing it from the VISITECT® CD4 LFA. This change was applied throughout the Methods and Results to improve clarity |
|  | Consider having all tables at the end and not within the text. | All tables have been moved to the end |
|  | Use of left over samples for Visitect was a methodological error as the manufacture instructs the sample to be run within 30minutes of sample collection, so overall not a very good output of this paper. | We thank the reviewer for raising this point. We would like to clarify that VISITECT® CD4 testing was not performed on leftover samples. Venous EDTA blood collected for each participant was pre‑specified to be split into two aliquots, with one aliquot used for PIMA® CD4 testing and the second aliquot independently used for VISITECT® CD4 LFA testing by the laboratory technician.  According to the manufacturer’s Instructions for Use, venous EDTA whole blood is an approved specimen type and may be used up to 24 hours post‑collection when stored at 2–30 °C. Therefore, short‑term refrigerated holding of EDTA samples prior to same‑day testing did not deviate from manufacturer recommendations and does not constitute a methodological error.  We have revised the Methods section to clarify specimen handling and remove any suggestion that leftover samples were used.  We have revised the Methods section to clarify specimen handling and remove any suggestion that leftover samples were used. |
|  | Line 234: Can this be moved down to where the figure is. Currently it feels misplaced. When its close to the figure it helps the reader better interprete the figure. | Figure 1 caption was moved down |
|  | Line 246: Would it be clearer to say The diagnostic accuracy of LFA ……is shown in table 1 instead of beginning with Cross tabulation……  Please include the sensitivity and specificity results along with the PPV and NPV as well as it’s a major study outcome. | We have revised the Results section to state explicitly that the diagnostic accuracy of the LFA is presented in Table 1 and have added a summary of the key diagnostic accuracy measures, including sensitivity, specificity, PPV, and NPV. The Results section has been updated accordingly. |
|  | Line 249.it would be good to summarize the results represented in supplementary table 1,right now we just have a summary of what it shows and not the result summary of the specificities and sensitivities, PPV/NPV. | Thank you for this suggestion. We have revised the Results section to explicitly summarize the sensitivity, specificity, PPV, and NPV values shown in Supplementary Table 1, rather than only referencing the table. The detailed results remain presented in the Supplementary Appendix |
|  | Line 311-320 is repeated in the discussion in line 321-331. | We have removed the repetition |
|  | In the discussion cite studies that have decentralized CD4 testing so we can compare how this study fits within the larger body of literature. Look up the Ugandan paper that validated visitect in a multisite study. | We have revised the Discussion to situate our findings within the broader literature on decentralized CD4 testing. We now cite evidence from a multisite study in Uganda validating the VISITECT® CD4 LFA, which demonstrated high sensitivity and acceptable specificity when implemented across diverse clinic settings. These findings are discussed alongside our results to highlight consistency of performance and feasibility of decentralized, lay‑provider–delivered CD4 testing. |
|  | Line 353-359 appear misplaced within that section of the discussion and the last sentences are a repetition, consider omitting. Are there other cost studies done for visitect? Please cite to make this section of the discussion more meaningful. | Thank you for this comment. We agree that the text in lines 353–359 was partially repetitive and not optimally placed. We have revised the Discussion to remove repetition and better integrate the cost considerations. In addition, we have strengthened this section by citing published programmatic evaluations that include costing and operational considerations for the VISITECT® CD4 test, thereby providing external context for our findings. The revised Discussion now cites relevant cost‑related literature and is more concise and informative. |
