# Supplementary material for: High performance of point-of-care rapid tests for advanced HIV disease diagnosis by lay providers in Malawi: Results from a prospective diagnostic accuracy study supporting decentralized advanced HIV disease screening
Source: PLoS One. 2026 Jun 18;21(6):e0340955. doi: 10.1371/journal.pone.0340955 (PMC13278447; doi:10.1371/journal.pone.0340955)
Supplement: Peer review history

# Peer review: Decision Letter 0

**DOI:** 10.1371/journal.pone.0340955.r001

**Authors:** Felix Bongomin

9 Feb 2026

PONE-D-25-68943

High performance of CD4 rapid testing by lay providers in Malawi: Results from a prospective diagnostic accuracy study supporting decentralized advanced HIV disease screening

PLOS One

Dear Dr. Thawani,

Thank you for submitting your manuscript to PLOS ONE. After careful consideration, we feel that it has merit but does not fully meet PLOS ONE’s publication criteria as it currently stands. Therefore, we invite you to submit a revised version of the manuscript that addresses the points raised during the review process.

Please submit your revised manuscript by Mar 26 2026 11:59PM. If you will need more time than this to complete your revisions, please reply to this message or contact the journal office at plosone@plos.org. When you're ready to submit your revision, log on to https://www.editorialmanager.com/pone/ and select the 'Submissions Needing Revision' folder to locate your manuscript file.

Please include the following items when submitting your revised manuscript:

- A letter that responds to each point raised by the academic editor and reviewer(s). You should upload this letter as a separate file labeled 'Response to Reviewers'.
- A marked-up copy of your manuscript that highlights changes made to the original version. You should upload this as a separate file labeled 'Revised Manuscript with Track Changes'.
- An unmarked version of your revised paper without tracked changes. You should upload this as a separate file labeled 'Manuscript'.

If you would like to make changes to your financial disclosure, please include your updated statement in your cover letter. Guidelines for resubmitting your figure files are available below the reviewer comments at the end of this letter.

If applicable, we recommend that you deposit your laboratory protocols in protocols.io to enhance the reproducibility of your results. Protocols.io assigns your protocol its own identifier (DOI) so that it can be cited independently in the future. For instructions see: https://journals.plos.org/plosone/s/submission-guidelines#loc-laboratory-protocols. Additionally, PLOS ONE offers an option for publishing peer-reviewed Lab Protocol articles, which describe protocols hosted on protocols.io. Read more information on sharing protocols at https://plos.org/protocols?utm_medium=editorial-email&utm_source=authorletters&utm_campaign=protocols.

We look forward to receiving your revised manuscript.

Kind regards,

Felix Bongomin, MB ChB, MSc, MMed, FECMM

Academic Editor

PLOS One

Journal requirements:

When submitting your revision, we need you to address these additional requirements.

1. Please ensure that your manuscript meets PLOS ONE's style requirements, including those for file naming. The PLOS ONE style templates can be found at

https://journals.plos.org/plosone/s/file?id=wjVg/PLOSOne_formatting_sample_main_body.pdf and

https://journals.plos.org/plosone/s/file?id=ba62/PLOSOne_formatting_sample_title_authors_affiliations.pdf

2. We note that there is identifying data in the Supporting Information file <Visitect_final_211225.xlsx>. Due to the inclusion of these potentially identifying data, we have removed this file from your file inventory. Prior to sharing human research participant data, authors should consult with an ethics committee to ensure data are shared in accordance with participant consent and all applicable local laws.

Data sharing should never compromise participant privacy. It is therefore not appropriate to publicly share personally identifiable data on human research participants. The following are examples of data that should not be shared:

-Name, initials, physical address

-Ages more specific than whole numbers

-Internet protocol (IP) address

-Specific dates (birth dates, death dates, examination dates, etc.)

-Contact information such as phone number or email address

-Location data

-ID numbers that seem specific (long numbers, include initials, titled “Hospital ID”) rather than random (small numbers in numerical order)

Data that are not directly identifying may also be inappropriate to share, as in combination they can become identifying. For example, data collected from a small group of participants, vulnerable populations, or private groups should not be shared if they involve indirect identifiers (such as sex, ethnicity, location, etc.) that may risk the identification of study participants.

Additional guidance on preparing raw data for publication can be found in our Data Policy (https://journals.plos.org/plosone/s/data-availability#loc-human-research-participant-data-and-other-sensitive-data) and in the following article: http://www.bmj.com/content/340/bmj.c181.long.

Please remove or anonymize all personal information, ensure that the data shared are in accordance with participant consent, and re-upload a fully anonymized data set. Please note that spreadsheet columns with personal information must be removed and not hidden as all hidden columns will appear in the published file.

If the reviewer comments include a recommendation to cite specific previously published works, please review and evaluate these publications to determine whether they are relevant and should be cited. There is no requirement to cite these works unless the editor has indicated otherwise.

[Note: HTML markup is below. Please do not edit.]

Reviewers' comments:

Reviewer's Responses to Questions

**Comments to the Author**

1. Is the manuscript technically sound, and do the data support the conclusions?

The manuscript must describe a technically sound piece of scientific research with data that supports the conclusions. Experiments must have been conducted rigorously, with appropriate controls, replication, and sample sizes. The conclusions must be drawn appropriately based on the data presented.

Reviewer #1: No

Reviewer #2: Yes

**********

2. Has the statistical analysis been performed appropriately and rigorously?

Reviewer #1: No

Reviewer #2: Yes

**********

3. Have the authors made all data underlying the findings in their manuscript fully available?

The PLOS Data policy requires authors to make all data underlying the findings described in their manuscript fully available without restriction, with rare exception (please refer to the Data Availability Statement in the manuscript PDF file). The data should be provided as part of the manuscript or its supporting information, or deposited to a public repository. For example, in addition to summary statistics, the data points behind means, medians and variance measures should be available. If there are restrictions on publicly sharing data—e.g. participant privacy or use of data from a third party—those must be specified.

Reviewer #1: Yes

Reviewer #2: Yes

**********

4. Is the manuscript presented in an intelligible fashion and written in standard English?

PLOS ONE does not copyedit accepted manuscripts, so the language in submitted articles must be clear, correct, and unambiguous. Any typographical or grammatical errors should be corrected at revision, so please note any specific errors here.

Reviewer #1: Yes

Reviewer #2: Yes

**********

5. Review Comments to the Author

Please use the space provided to explain your answers to the questions above. You may also include additional comments for the author, including concerns about dual publication, research ethics, or publication ethics. (Please upload your review as an attachment if it exceeds 20,000 characters)

Reviewer #1: The authors are attempting to address an important subject but they are several weaknesses with this report. To start with there are several gramma/ typographical errors that could have been taken care of before submission. Additionally, the name of the LFA being evaluated is only disclosed much later in the methodology section and is a bit surprising when it is finally mentioned as Visitect, yet through out the abstract or the introduction, there is no reference to visitect. There are some publications on visitect that should have been referenced, the authors then needed to make the case for what is different in the work that is being presented from previous evaluations.

Abstract: There is ambiguity. The name of the tool being evaluated is not mentioned, additionally, in the abstract results section, one then realised that this evaluation extends beyond CD4 LFA. The authors should consider revising the title to take care of the additional

Additional comments

Line 68 : Large proportion is ambiguous, gives impression more PLHIV are AHD than are controlled, yet this is not the case? Needs revision.

Line 74, can abbreviate PLHIV here

Line 79-81 should be referenced

Line 93, does this test have a name at all or it is just CD4 LFA?

line 97- second objective comes as a bit of a surprise, the title is silent, consider revising the title a

line 126, what quantitative NPOC device is this? It is important to name it, so readers can reference

Line 133; Tests used: This needs to been mentioned earlier, until one gets to this line does one realise that the LFA being evaluated is visitect. See earlier comment on the same. Adn no reference to other works on visitect. There are several publications on visitect performance, some reference needs to be made to these in the introductory section, then what is this study has done differently to deserve being published

Need to be clear on :

1. CD4 test LFA being evaluated? Name and manufacturer, has it been evaluated before?

2. Which test is the reference standard, is it PIMA semi quantitative or Visitect?

3. If CD4 LFA being evaluated is not visitect, clearly mention in the methodology that CD4 LFA is also being compared against visitect.

line158: Name the assay

Line 167, name the near point of care to remove ambiguity

Line 169 to 174, needs to be clear if CrAg was performed on venous blood collected for NPOC, or finger stick blood was collected. What is sample type recommended by the manufacturer. For urine lam was a fresh sample collected? How long before testing ?

Line 173 to 175- Why storage, why not test immediately. What is the sample type recommended for CD4 LFA? Refer to the SOP for visitect and clarify that using venous blood did not deviate from the recommended SOP. And is storage of samples the current practice? Initially, one gets the impression that comparison of CD4 LFA test by HDA, is comparing to qualified health staff using the exact methodology , then later one learns that different samples are used, and there is storage

179 to 181; Clarity? which specific results

Line 186 to 188, review whole sentence and revise as needed, it is difficult to read due to gramma/ typos

Line 190. Clarify, two sensitivity analysis, but I only see one analysis below?

Line 199: does this mean the sample was not adequate for accuracy evaluation ( sensitivity and Specificity), yet accuracy is the main objective for this evaluation and not reproducibility?

Line 247- predictor or predictive, and why focus on 28% simulated prevalence , why not report actual findings from this study?

Line 321 is repetition. review and remedy as needed

403- Any comment on Sample size, was it adequate for accuracy evaluation?

line 410, brief or minimal ?

References don't seem to follow the seem to follow the same format through out.

Reviewer #2: Sent earlier

**********

6. PLOS authors have the option to publish the peer review history of their article (what does this mean?). If published, this will include your full peer review and any attached files.

If you choose “no”, your identity will remain anonymous but your review may still be made public.

**Do you want your identity to be public for this peer review?**  For information about this choice, including consent withdrawal, please see our Privacy Policy.

Reviewer #1: No

Reviewer #2: No

**********

[NOTE: If reviewer comments were submitted as an attachment file, they will be attached to this email and accessible via the submission site. Please log into your account, locate the manuscript record, and check for the action link "View Attachments". If this link does not appear, there are no attachment files.]

To ensure your figures meet our technical requirements, please review our figure guidelines: https://journals.plos.org/plosone/s/figures

You may also use PLOS’s free figure tool, NAAS, to help you prepare publication quality figures: https://journals.plos.org/plosone/s/figures#loc-tools-for-figure-preparation.

NAAS will assess whether your figures meet our technical requirements by comparing each figure against our figure specifications.

# Author comment: Author response to Decision Letter 1

**DOI:** 10.1371/journal.pone.0340955.r002

30 Apr 2026

Manuscript ID: PONE-D-25-68943

Title: High performance of point-of-care rapid tests for advanced HIV disease diagnosis by lay providers in Malawi: Results from a prospective diagnostic accuracy study supporting decentralized advanced HIV disease screening

Response to the Academic Editor

Dear Academic Editor,

We thank you and the reviewers for the careful evaluation of our manuscript and for the constructive and insightful comments provided. We have revised the manuscript extensively in response to all points raised.

To facilitate review of the revisions, we provide below a detailed, point by point response in which each reviewer comment is reproduced verbatim followed by our corresponding response and a description of the changes made. All revisions have been incorporated into the revised manuscript.

In summary, the key revisions include:

• Explicit identification of the VISITECT® CD4 lateral flow assay at first mention in the Abstract, Introduction, and Methods;

• Inclusion of relevant prior literature on VISITECT® CD4 performance and a clearer articulation of the novel contribution of this study;

• Improved clarity regarding specimen types, handling procedures, and reference standards;

• Strengthened presentation and interpretation of diagnostic accuracy results, including justification of prevalence assumptions used for predictive value estimation;

• Reorganization and refinement of the Results and Discussion sections to remove redundancy and improve readability;

• Comprehensive correction of grammatical, typographical, and formatting inconsistencies throughout the manuscript.

We believe that these revisions have substantially strengthened the manuscript and addressed all concerns raised by the reviewers. We appreciate the opportunity to revise our work and thank you again for your time and consideration.

Sincerely,

Agness Thawani

Corresponding Author

Reviewer 1 – Point by Point Responses

Comment 1

There are several grammatical/typographical errors that could have been taken care of before submission.

Response:

The manuscript has been carefully revised, and grammatical, typographical, and formatting errors have been corrected throughout.

Comment 2

The name of the LFA being evaluated is only disclosed much later in the methodology section… there is no reference to VISITECT in the abstract or introduction.

Response:

We agree. The Abstract and Introduction have been revised to explicitly identify the VISITECT® CD4 lateral flow assay at first mention, ensuring the diagnostic assay under evaluation is clearly stated upfront.

Comment 3

There are publications on VISITECT that should have been referenced; the authors need to clarify what is novel about this work.

Response:

The Introduction has been revised to cite prior VISITECT® CD4 evaluations and to clearly state the added value of the present study: namely, assessment of diagnostic accuracy, feasibility, and cost when VISITECT® CD4 testing is performed by lay providers in routine programmatic settings.

Comment 4

Abstract: The name of the tool being evaluated is not mentioned.

Response:

The Abstract now explicitly identifies the VISITECT® CD4 lateral flow assay.

Comment 5

The abstract results suggest that the evaluation extends beyond CD4 LFA.

Response:

The Abstract has been clarified to state upfront that the evaluation includes VISITECT® CD4 testing as well as serum cryptococcal antigen (CrAg) and urine TB-LAM diagnostics as part of advanced HIV disease screening.

Comment 6

The title should reflect the additional tests evaluated.

Response:

The Title has been revised to reflect evaluation of advanced HIV disease diagnostics, with CD4 testing as the entry point assay and reflex CrAg and TB LAM testing, without overstating test specific accuracy claims.

Comment 7

Line 68: “Large proportion” is ambiguous.

Response:

This phrase has been replaced with a quantified statement indicating that approximately one quarter to one third of people presenting to care have advanced HIV disease, removing ambiguity.

Comment 8

Line 74: Can abbreviate PLHIV here.

Response:

“People living with HIV (PLHIV)” is now defined at first use, and the abbreviation is used consistently thereafter.

Comment 9

Line 79–81 should be referenced.

Response:

Appropriate references have been added to support statements regarding CD4 testing and opportunistic infection risk.

Comment 10

Line 93: Does this test have a name?

Response:

The VISITECT® CD4 assay is now explicitly named wherever first referenced.

Comment 11

Line 97: Second objective is surprising given the title.

Response:

The Title has been revised to encompass all diagnostics assessed.

Comment 12

Line 126: What quantitative near POC device is this?

Response:

The PIMA® CD4 device is now named explicitly.

Comment 13

Line 133: Clarify tests evaluated, reference standard, and prior VISITECT work.

Response:

We clarified that:

1. The index test is VISITECT® CD4 LFA (Omega Diagnostics);

2. The reference standard is PIMA® CD4;

3. Prior VISITECT® studies are now cited;

4. The novelty lies in evaluation by lay providers under routine conditions, including feasibility and cost.

Comment 14

Line 158: Name the assay.

Response:

The assay is now explicitly identified as VISITECT® CD4.

Comment 15

Line 167: Name the near POC device.

Response:

“NPOC” has been replaced with PIMA® CD4.

Comment 16

Lines 169–174: Clarify specimen types and timing for CrAg and TB LAM.

Response:

The Methods now specify:

• Finger prick capillary blood for serum CrAg (manufacturer approved);

• Fresh urine samples tested immediately for TB LAM.

Comment 17

Lines 173–175: Clarify storage, specimen type, and comparability.

Response:

We clarified that VISITECT® CD4 permits both capillary and venous EDTA blood. Venous EDTA blood was used for laboratory testing to allow paired testing with PIMA® CD4 and minimize repeat fingerpricks. Storage and timing complied with manufacturer SOPs. Distinctions between point of care and laboratory workflows are now clearly described.

Comment 18

Lines 179–181: Which results were shared with clinicians?

Response:

We clarified that only nurse performed standard of care results (PIMA® CD4, CrAg, TB LAM) were shared for clinical management.

Comment 19

Lines 186–188: Sentence is difficult to read.

Response:

The sentence has been revised for clarity.

Comment 20

Line 190: Two sensitivity analyses mentioned but only one described.

Response:

We revised the text to describe one sensitivity analysis, with the Gart and Buck method now described as an exploratory correction rather than a second sensitivity analysis.

Comment 21

Line 199: Sample size adequacy for accuracy evaluation?

Response:

We clarified that sample size assumptions ensured sufficient distribution across CD4 categories and were adequate for estimating sensitivity and specificity, which remained the primary objective.

Comment 22

Line 247: “Predictor” vs “predictive”; why simulate 28% prevalence?

Response:

“Predictor” was corrected to “predictive.” Observed prevalence (40%) is used for primary PPV/NPV estimates; 28% prevalence reflects national AHD prevalence in Malawi to enhance generalizability.

Comment 23

Line 321: Repetition.

Response:

Repetition removed.

Comment 24

Lines 403–410: Sample size adequacy; clarify “brief/minimal.”

Response:

We clarified that the target sample of 308 provided acceptable precision, yielding >100 participants with CD4 ≤200 cells/µL. Ambiguous wording has been revised.

Comment 25

References inconsistent.

Response:

All references have been standardized to journal format.

Reviewer 2 – Point by Point Responses

Comment 1

Define AHD eligibility and describe clinic burden.

Response:

Advanced HIV disease is now explicitly defined according to Malawi national guidelines (CD4 ≤200 cells/µL). The Lighthouse ART Clinic is described as a high volume referral site, with details on patient load and nursing workload included in the Study Setting.

Comment 2

Use “PIMA” rather than “near POC device.”

Response:

Throughout the manuscript, the reference assay is now consistently referred to as PIMA® CD4.

Comment 3

Move all tables to the end.

Response:

All tables have been moved to the end of the manuscript.

Comment 4

Use of leftover samples for VISITECT is a methodological error.

Response:

We clarified that responses were not leftover samples. EDTA blood was prospectively collected and split as per protocol. Manufacturer IFU allows EDTA blood use up to 24 hours post collection, and specimen handling complied fully with SOPs. The Methods have been revised to remove any ambiguity.

Comment 5

Line 234: Move text closer to the figure.

Response:

Figure 1 caption has been repositioned accordingly.

Comment 6

Line 246: Rephrase and include sensitivity/specificity.

Response:

The Results now explicitly state that diagnostic accuracy is presented in Table 1 and summarize sensitivity, specificity, PPV, and NPV.

Comment 7

Line 249: Summarize Supplementary Table 1 results.

Response:

The Results now explicitly summarize sensitivity, specificity, PPV, and NPV from Supplementary Table 1.

Comment 8

Lines 311–320 repeated later.

Response:

Repetitive text has been removed.

Comment 9

Cite decentralized CD4 studies, including Ugandan VISITECT study.

Response:

The Discussion now cites multisite Ugandan validation studies and situates our findings within the broader decentralized CD4 testing literature.

Comment 10

Lines 353–359 misplaced and repetitive; cite VISITECT cost studies.

Response:

This section has been streamlined, repositioned, and strengthened by citing published programmatic and cost evaluations of VISITECT® CD4 testing.

Attachment Submitted filename: Response to Reviewers.docx

# Peer review: Decision Letter 1

**DOI:** 10.1371/journal.pone.0340955.r003

**Authors:** Felix Bongomin

14 May 2026

High performance of point-of-care rapid tests for advanced HIV disease diagnosis by lay providers in Malawi: Results from a prospective diagnostic accuracy study supporting decentralized advanced HIV disease screening

PONE-D-25-68943R1

Dear Dr. Thawani,

We’re pleased to inform you that your manuscript has been judged scientifically suitable for publication and will be formally accepted for publication once it meets all outstanding technical requirements.

Within one week, you’ll receive an e-mail detailing the required amendments. When these have been addressed, you’ll receive a formal acceptance letter and your manuscript will be scheduled for publication.

An invoice will be generated when your article is formally accepted. Please note, if your institution has a publishing partnership with PLOS and your article meets the relevant criteria, all or part of your publication costs will be covered. Please make sure your user information is up-to-date by logging into Editorial Manager at Editorial Manager® and clicking the ‘Update My Information' link at the top of the page. For questions related to billing, please contact billing support.

If your institution or institutions have a press office, please notify them about your upcoming paper to help maximize its impact. If they’ll be preparing press materials, please inform our press team as soon as possible -- no later than 48 hours after receiving the formal acceptance. Your manuscript will remain under strict press embargo until 2 pm Eastern Time on the date of publication. For more information, please contact onepress@plos.org.

Kind regards,

Felix Bongomin, MB ChB, Ph.D., FECMM

Academic Editor

PLOS One

Additional Editor Comments (optional):

Reviewers' comments:

Reviewer's Responses to Questions

**Comments to the Author**

1. If the authors have adequately addressed your comments raised in a previous round of review and you feel that this manuscript is now acceptable for publication, you may indicate that here to bypass the “Comments to the Author” section, enter your conflict of interest statement in the “Confidential to Editor” section, and submit your "Accept" recommendation.

Reviewer #2: All comments have been addressed

**********

2. Is the manuscript technically sound, and do the data support the conclusions?

The manuscript must describe a technically sound piece of scientific research with data that supports the conclusions. Experiments must have been conducted rigorously, with appropriate controls, replication, and sample sizes. The conclusions must be drawn appropriately based on the data presented.

Reviewer #2: Yes

**********

3. Has the statistical analysis been performed appropriately and rigorously?

Reviewer #2: Yes

**********

4. Have the authors made all data underlying the findings in their manuscript fully available?

The PLOS Data policy requires authors to make all data underlying the findings described in their manuscript fully available without restriction, with rare exception (please refer to the Data Availability Statement in the manuscript PDF file). The data should be provided as part of the manuscript or its supporting information, or deposited to a public repository. For example, in addition to summary statistics, the data points behind means, medians and variance measures should be available. If there are restrictions on publicly sharing data—e.g. participant privacy or use of data from a third party—those must be specified.

Reviewer #2: Yes

**********

5. Is the manuscript presented in an intelligible fashion and written in standard English?

PLOS ONE does not copyedit accepted manuscripts, so the language in submitted articles must be clear, correct, and unambiguous. Any typographical or grammatical errors should be corrected at revision, so please note any specific errors here.

Reviewer #2: Yes

**********

6. Review Comments to the Author

Please use the space provided to explain your answers to the questions above. You may also include additional comments for the author, including concerns about dual publication, research ethics, or publication ethics. (Please upload your review as an attachment if it exceeds 20,000 characters)

Reviewer #2: Great job team malawi. The manuscript reads well and clearly outlines what was done and how the results were obtained and how they apply to AHD. The team has done an excelent job in responding to the comments.

Minor comments:

1. Please describe in the methods who the HDAs were,what level of education, were they HIV positive peers, what were the minimum requirements needs to be recruited as an HDA. This will help inform implementation of this intervention in other settings.

2. Line 188: add hyphen in the word re engagement.

3. Just sharing our Ugandan experience, Samples that were not run within 30 minutes of bleeding despite what the insert states. This has been high lighted well in the write up and is a significant implementation issue.

**********

7. PLOS authors have the option to publish the peer review history of their article (what does this mean?). If published, this will include your full peer review and any attached files.

If you choose “no”, your identity will remain anonymous but your review may still be made public.

**Do you want your identity to be public for this peer review?**  For information about this choice, including consent withdrawal, please see our Privacy Policy.

Reviewer #2: No

**********

# Editor report: Acceptance letter

**DOI:** 10.1371/journal.pone.0340955.r004

**Authors:** Felix Bongomin

PONE-D-25-68943R1

PLOS One

Dear Dr. Thawani,

I'm pleased to inform you that your manuscript has been deemed suitable for publication in PLOS One. Congratulations! Your manuscript is now being handed over to our production team.

At this stage, our production department will prepare your paper for publication. This includes ensuring the following:

* All references, tables, and figures are properly cited

* All relevant supporting information is included in the manuscript submission,

* There are no issues that prevent the paper from being properly typeset

You will receive further instructions from the production team, including instructions on how to review your proof when it is ready. Please keep in mind that we are working through a large volume of accepted articles, so please give us a few days to review your paper and let you know the next and final steps.

Lastly, if your institution or institutions have a press office, please let them know about your upcoming paper now to help maximize its impact. If they'll be preparing press materials, please inform our press team within the next 48 hours. Your manuscript will remain under strict press embargo until 2 pm Eastern Time on the date of publication. For more information, please contact onepress@plos.org.

You will receive an invoice from PLOS for your publication fee after your manuscript has reached the completed accept phase. If you receive an email requesting payment before acceptance or for any other service, this may be a phishing scheme. Learn how to identify phishing emails and protect your accounts at https://explore.plos.org/phishing.

If we can help with anything else, please email us at customercare@plos.org.

Thank you for submitting your work to PLOS ONE and supporting open access.

Kind regards,

PLOS ONE Editorial Office Staff

on behalf of

Dr. Felix Bongomin

Academic Editor

PLOS One
